# Hidradenitis Suppurativa Is Associated with an Increased Risk of Adverse Cardiac Events and All-Cause Mortality

**DOI:** 10.3390/jcm14041110

**Published:** 2025-02-09

**Authors:** Thomas Z. Rohan, Ramsay Hafer, Teresa Duong, Rishob Dasgupta, Sherry Yang

**Affiliations:** 1 Sidney Kimmel Medical College, Thomas Jefferson University, Philadelphia, PA 19107, USA; tzr002@students.jefferson.edu (T.Z.R.);; 2 Department of Dermatology and Cutaneous Biology, Thomas Jefferson University, Philadelphia, PA 19107, USA

**Keywords:** hidradenitis suppurativa, cardiovascular disease, psoriasis, inflammatory skin diseases

## Abstract

**Background**: Hidradenitis suppurativa (HS) has been previously associated with greater rates of major adverse cardiac events (MACEs) compared to the general population. This study aims to better elucidate the association between HS, MACEs, and other cardiovascular diseases. **Methods**: We utilized TriNetX, a global database of electronic health records, to conduct a retrospective cohort study. HS patients were matched on demographic and cardiovascular disease risk factors to both healthy and psoriasis groups as controls. **Results**: After adjusting for cardiovascular disease risk factors, HS patients had a relative risk (RR) (95% CI) of 2.06 (1.83–2.32) for myocardial infarction, 1.62 (1.44–1.82) for ischemic stroke, 2.21 (2.04–2.40) for heart failure, 1.95 (1.84–2.07) for MACEs, and 2.57 (2.34–2.83) for all-cause mortality compared to healthy controls. When comparing HS patients to matched psoriasis controls, HS patients had an RR of 1.31 (1.17–1.47) for myocardial infarction, 1.04 (0.93–1.16) for ischemic stroke, 1.24 (1.15–1.34) for heart failure, 1.16 (1.09–1.22) for MACEs, and 1.38 (1.27–1.5) for all-cause mortality. Herein, we demonstrate that patients with HS have increased rates of all cardiovascular diseases investigated when compared to healthy and psoriasis controls, even after adjusting for cardiovascular disease risk factors. **Conclusions**: These findings highlight the potential benefit in screening for and managing modifiable cardiovascular risk factors in HS patients.

## 1. Background

Hidradenitis suppurativa (HS) is a chronic inflammatory skin condition characterized by painful, draining abscesses. While the exact pathophysiology is poorly understood, HS likely begins with follicular occlusion. This obstruction then causes the rupture of contents into the subdermal space, with a resulting inflammatory cascade ensuing [1]. Prior works have shown the elevated expression of inflammatory cytokines, such as interleukin (IL)-17, IL-12, and tumor necrosis factor-α (TNF-α), within HS lesions [2,3,4]. In addition, circulating markers of systemic inflammation, including serum TNF-α and C-reactive protein, appear higher in HS than other inflammatory skin disorders [5].

Inflammation likely plays a large role in atherogenesis, triggering its early phases through endothelial dysfunction and increased permeability to lipoproteins. Furthermore, in an inflammatory environment, lipoprotein metabolism is shifted from large- and medium-size to small low-density lipoprotein cholesterol, which have been associated with an elevated risk of arterial disease due to their increased persistence in the blood and tendency to enter the arterial wall [6,7]. Because of the close relationship between inflammation and poor cardiovascular outcomes, the association of inflammatory dermatologic conditions and said outcomes warrant investigation.

Systemic inflammatory responses, such as those in inflammatory bowel disease and inflammatory polyarthropathies, have been associated with greater rates of cardiovascular (CV) mortality and major adverse cardiac events (MACEs), such as cardiac arrest, myocardial infarction (MI), and stroke [8,9,10]. Other chronic inflammatory dermatologic conditions, such as atopic dermatitis and psoriasis, have also been linked to MACEs [11,12,13]. One proposed mechanism for the association with dermatologic conditions is inflammatory cytokines from surface-level lesions leaking into dilated and fenestrated local capillaries, joining the systemic circulation [14]. Therefore, more severe or prolonged levels of inflammation may correlate with the earlier onset or higher prevalence of MACEs. With cardiovascular disease continuing to be the leading cause of morbidity and mortality worldwide, the identification of at-risk patients is a high priority for better health outcomes [15].

Studies have previously demonstrated the high prevalence of metabolic risk factors for severe cardiovascular disease, such as hypertension, diabetes mellitus, and dyslipidemias, in HS patients [16]. This relationship has also been established for psoriasis patients [14,17,18,19]. Therefore, studies investigating a potential independent relationship between both HS and psoriasis on cardiovascular disease should match for these risk factors to mitigate potential bias introduced by these other comorbidities. While recent studies have found an increased risk of CV disease in HS patients compared to healthy controls, few have compared HS and psoriasis patients or have ultimately displayed conflicting results [20,21,22,23]. Other studies investigating this relationship are primarily based on single-center analyses, contain small sample sizes, have a limited scope of outcomes, or utilize potentially inadequate matching criteria [20,21,22,23,24].

To further analyze the relationship between HS and MACEs, we conducted a retrospective cohort study utilizing TriNetX, a global health research network which provides access to a far more diverse and robust patient population. We have also investigated the prevalence and relative risk of primary CV disease risk factors, as well as analyzing outcomes both before and after matching to controls for said risk factors.

## 2. Methods

### 2.1. Patient Population

At the time of this study on 10 December 2024, the TriNetX analytics subset of patients (age > 18 years) contained over 102 million patients across 69 healthcare organizations in the United States collaborative network (TriNetX Inc., Cambridge, MA, USA). The platform includes deidentified patient information on basic demographics, diagnoses, lab results, procedures, and medications from the inpatient, outpatient, and emergency department settings. TriNetX is certified to the standard defined in Section §164.514(a) of the Health Insurance Portability and Accountability Act (HIPAA) Security Rule. All patient data are deidentified and are therefore exempt from Institutional Review Board approval.

Our study population consisted of patients 18 years of age or older with HS coded on at least two separate occasions. Healthy controls were defined as patients ≥18 years with at least two separate encounters for general adult medical examination and no history of HS or psoriasis. Lastly, the psoriasis cohort included patients ≥18 years of age with at least two International Classification of Diseases, 10th edition (ICD-10) codes of psoriasis and no concurrent or prior history of HS. To allow enough time for outcomes of interest to develop between the index event and the final analysis, at least one of the codes had to occur before 31 December 2022 for all three groups. The index event for each population was the first coding for HS, general medical examination period, or psoriasis that occurred within the specified time range. Patients from all three populations were excluded if they had a history of major adverse cardiac events (defined as cardiac arrest, stroke, or MI), venous thrombosis, or autoimmune disease prior to the index event to analyze the temporal relationship between each respective condition and relevant outcomes. Any outcome occurring between the index event and the date of final analysis (10 December 2024) was included in the analysis.

### 2.2. Covariates

To evaluate the prevalence of primary cardiovascular disease risk factors (hypertension, diabetes mellitus, dyslipidemia), we performed 1:1 propensity score matching between the HS and control cohorts based on current age, sex, race, body mass index (BMI), nicotine dependence, and ethnicity. When evaluating the risk of more severe cardiovascular disease and MACEs, we adjusted our analysis to also match cohorts on alcohol use disorder (F10), nicotine dependence (F17), disorders of lipoprotein metabolism and other lipidemias (E78), liver disease (K70–77), diabetes mellitus (E08–13), chronic lower respiratory diseases (J40–J4A), immunological agents/suppressants (IM000 and IM600), and primary hypertension (I10). The TriNetX system employs input matrices containing user-identified covariates and conducts logistic regression analysis to generate propensity scores for individual subjects. To mitigate bias stemming from nearest neighbor algorithms, TriNetX introduces randomness by randomizing the order of rows.

### 2.3. Outcomes

When evaluating for general cardiovascular disease risk factors, outcomes of interest were considered to be primary hypertension (I10), diabetes mellitus (E08–13), and/or dyslipidemia (E78.0–78.5). When matching for the presence of these risk factors, primary endpoints consisted of myocardial infarction (MI) (I21 and I22), ischemic cerebrovascular accident (CVA) (I63), and all-cause mortality. Secondary endpoints included heart failure (HF) (I50), hemorrhagic CVA (I61 and I62), venous thromboembolisms (VTE) (I26 and I82), essential hypertension (HTN) (I10), cardiac arrest (I46), coronary artery disease (CAD) (I25.1), peripheral arterial disease (PAD) (I70), and major adverse cardiovascular events (MACEs), which are a summation of heart failure (HF), myocardial infarction (MI), cardiac arrest, and ischemic and/or hemorrhagic CVA (I21, I22, I46, I50, I61–63).

### 2.4. Statistical Analysis

After the optimization of the two cohorts, adverse outcomes were identified and counted with ICD-10-CM codes that were entered anytime between the onset of the index event and the date of data retrieval (10 December 2024). We also stratified risk by sex and by current age for subgroup analysis between HS and control cohorts. All statistical analysis was performed on the TriNetX platform. For the univariate analysis, continuous variables (age and BMI) were compared using independent samples *t*-tests, and binary variables were compared using chi-squared tests. The risk assessment was performed by calculating risk ratios, which are proportions representing the risk of developing an outcome in hidradenitis suppurativa divided by the risk of developing the same outcome in the control population.

## 3. Results

The database contained 218,747 total HS patients; 7817 were excluded due to being younger than 18 years of age, 42,682 were excluded due to having cardiovascular or autoimmune disease diagnosed prior to HS, and 104,691 more were excluded due to only having one diagnosis of HS in the system. There were 63,557 patients that met final HS inclusion criteria and 61,200 included after matching. The baseline characteristics for both analyses are included in Table 1. The mean age of the patients was 42.9 ± 13.4; 46,289 (75.6%) were women. The mean follow-up time was 4.6 ± 2.6 years for the control cohort and 5.3 ± 3.1 years for the HS cohort.

Hidradenitis suppurativa patients had a higher incidence of primary cardiovascular risk factors compared to healthy controls. They faced a 19% increased risk of essential hypertension (relative risk (RR): 1.19; 95% CI: 1.17–1.20; *p* < 0.0001), 136% increase in risk of atherosclerotic disease (RR 2.36; 95% CI: 2.12–2.64; *p* < 0.0001), and 82% increase in risk of diabetes mellitus (RR 1.82; 95% CI: 1.77–1.87; *p* < 0.0001).

**Figure 1:**
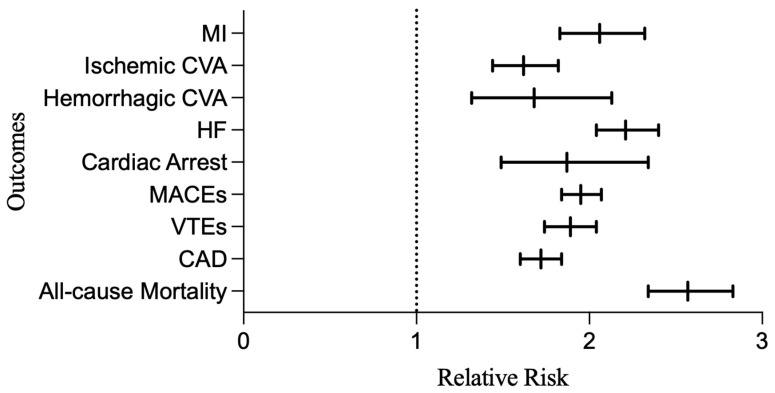
Relative risk of cardiovascular outcomes in HS vs. healthy patients. Abbreviations: HS, hidradenitis suppurativa; MI, myocardial infarction; CVA, cerebrovascular accident; HF, heart failure; MACEs, major adverse cardiac events; VTEs, venous thromboembolisms; CAD, coronary artery disease.

After matching for cardiovascular disease risk factors, there were 850 (1.39% of the HS cohort) MIs, 717 (1.17%) ischemic CVAs, 178 (0.29%) hemorrhagic CVAs, 1850 (3.02%) HFs, 217 (0.36%) cardiac arrests, 1708 (2.79%) VTEs, 2970 (4.86%) MACEs, 2136 (3.49%) CADs, and 1473 (2.4%) any-cause deaths in the HS population. After adjusting for cardiovascular disease risk factors, HS patients faced a relative risk (95% CI; *p*-value) of 2.06 (1.83–2.32; *p* < 0.0001) for MI, 1.62 (1.44–1.82; *p* < 0.0001)) for ischemic CVA, 1.68 (1.32–2.13; *p* < 0.0001) for hemorrhagic CVA, 2.21 (2.04–2.40; *p* < 0.0001) for HF, 1.87 (1.49–2.34; *p* < 0.0001) for cardiac arrest, 1.89 (1.74–2.04; *p* < 0.0001) for VTE, 1.72 (1.60–1.84; *p* < 0.0001) for CAD, 1.95 (1.84–2.07; *p* < 0.0001) for MACEs, and 2.57 (2.34–2.83; *p* < 0.0001) for all-cause mortality compared to healthy controls (Table 2, Figure 1).

**Figure 2:**
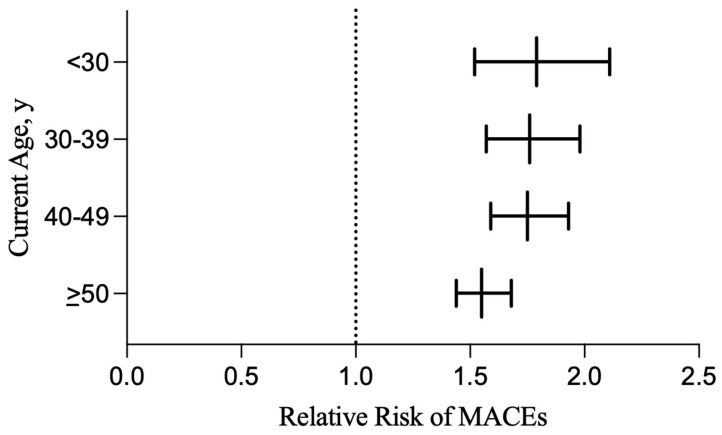
Relative risk of cardiovascular outcomes in HS vs. healthy patients, stratified by current age. Abbreviations: HS, hidradenitis suppurativa; MACEs, major adverse cardiac events.

When stratifying risk by sex, female HS patients demonstrated an elevated risk of ischemic strokes (RR: 1.62; 95% CI, 1.38–1.89; *p* < 0.0001) and cardiac arrest (RR: 1.49; 95% CI 1.11–1.99; *p* = 0.0071), while males did not have an elevated risk of these outcomes (Table 3). Risk profiles were otherwise similar between males and females, except for males having a significantly higher risk of all-cause mortality (Table 3). When stratifying risk of MACEs by patients’ current age, there were significant differences in each age group. The risk of MACEs in those currently younger than 30 was 1.79 (1.52–2.11; *p* < 0.0001), 1.76 (1.57–1.98; *p* < 0.0001) in those 30–39, 1.75 (1.59–1.93; *p* < 0.0001) in those 40–49, and 1.55 (1.44–1.68; *p* < 0.0001) in those currently ≥50 years of age (Figure 2).

**Figure 3:**
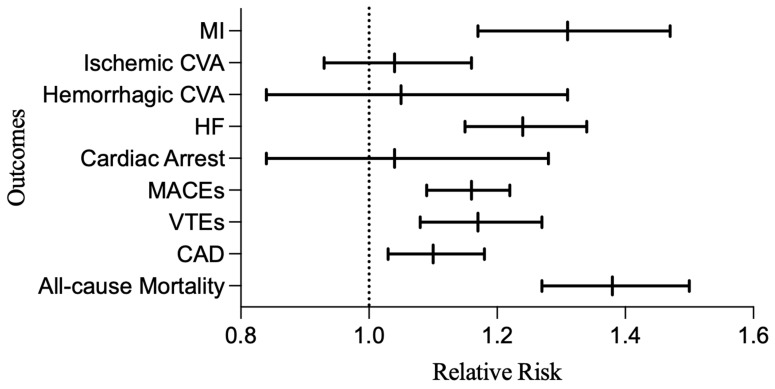
Relative risk of cardiovascular outcomes in HS vs. psoriasis patients. Abbreviations: HS, hidradenitis suppurativa; MI, myocardial infarction; CVA, cerebrovascular accident; HF, heart failure; MACEs, major adverse cardiac events; VTEs, venous thromboembolisms; CAD, coronary artery disease.

In a secondary analysis with psoriasis patients as controls, there were 48,163 HS and psoriasis patients included after matching for demographics and risk factors for cardiovascular disease (Table 4). Relative risks in patients with HS were 1.31 (1.17–1.47; *p* < 0.0001) for MI, 1.04 (0.93–1.16; *p* = 0.9557) for ischemic CVAs, 1.05 (0.84–1.31; *p* = 0.6847) for hemorrhagic CVAs, 1.24 (1.15–1.34; *p* < 0.0001) for HF, 1.04 (0.84–1.28; *p* = 0.7441) for cardiac arrest, 1.17 (1.08–1.27; *p* < 0.0001) for VTEs, 1.20 (1.17–1.23; *p* < 0.0001) for HTN, 1.10 (1.03–1.18; *p =* 0.0025) for CAD, 1.16 (1.09–1.22; *p* < 0.0001) for MACEs, and 1.38 (1.27–1.5; *p* < 0.0001) for all-cause mortality (Table 5, Figure 3).

## 4. Discussion

After adjusting for potential confounding factors, such as hypertension, BMI, and age, we found a significantly increased risk of major adverse cardiac events, myocardial infarction, ischemic stroke, hemorrhagic stroke, heart failure, venous thromboembolism, coronary artery disease, cardiac arrest, and all-cause mortality in patients with HS compared to healthy controls. Each of these risks also remained significant when comparing patients with HS to psoriasis controls regardless of patient sex, except for male HS patients not facing a statistically significant difference in ischemic stroke or cardiac arrest compared to male controls. Finally, the increase in relative risk was present regardless of current age stratification. These findings strongly suggest that HS independently increases cardiovascular disease risk and all-cause mortality for patients.

The previous literature suggests that the hyperkeratinization of the follicular infundibulum and subsequent follicular occlusion and rupture in HS patients is associated with the inflammatory state observed in HS [4]. Specifically, this process may induce systemic inflammation by increasing serum levels of cytokines like IL-17, TNF-α, and IL-1β [2,4,25]. Similarly, HS patients have been found to have higher systemic inflammatory markers (C-reactive protein, circulating leukocyte counts) and pro-inflammatory cytokine levels (TNF-α) than other chronic inflammatory diseases like psoriasis [4,5]. Another study assessed 92 inflammatory protein biomarkers and found that the inflammatory proteome of HS skin is significantly more expansive than that of psoriasis, suggesting that inflammation in HS skin may be more severe than in psoriasis [26].

Inflammation is thought to play a large role in atherogenesis, triggering its early phases through increasing endothelial permeability to lipoproteins and shifting lipoprotein metabolism from large- and medium-size to smaller, low-density lipoprotein cholesterol. These processes have been associated with an elevated risk of arterial disease due to their increased persistence in the blood and tendency to enter the arterial wall [6,7]. The influence of inflammatory actors may explain the association of HS and other inflammatory disorders with accelerated atherogenesis [27].

The previous literature suggests that the hyperkeratinization of the follicular infundibulum and subsequent follicular occlusion and rupture in HS patients is associated with the inflammatory state observed in HS [4]. Specifically, this process may induce systemic inflammation by increasing serum levels of cytokines like IL-17, TNF-ɑ, and IL-1β [2,4,25]. Additionally, HS patients have been found to have higher systemic inflammatory markers (C-reactive protein, circulating leukocyte counts) and pro-inflammatory cytokine levels (TNF-α) than other chronic inflammatory diseases like psoriasis [4,5]. This could explain the elevated risk seen in HS patients even when using psoriasis patients as a control population.

Prior works have also observed an association between other states of chronic inflammation, such as psoriasis, systemic lupus erythematosus, and rheumatoid arthritis, with cardiovascular (CV) disease and mortality [12,28]. Psoriasis in particular has been well described in the literature as a risk factor for CV disease [14,18,19]. While recent studies have found an increased risk of CV disease in HS patients compared to healthy controls, few have compared HS and psoriasis patients [20,21,22,23]. Such studies have displayed conflicting results. In a population-based cohort study of Danish individuals, the risk of myocardial infarction, ischemic stroke, MACEs, and all-cause mortality in patients with HS was similar to that of patients with severe psoriasis, but the risk of CV-associated death was significantly higher in patients with HS [20]. In another single-center retrospective cohort study of HS patients, the risk of stroke, peripheral arterial disease, and heart failure were not significantly different between HS and psoriasis patients, while risk of coronary artery disease was significantly higher in patients with HS [29]. However, our study revealed that HS patients carried increased risks of MACEs, myocardial infarction, heart failure, venous thromboembolism, coronary artery disease, and all-cause mortality compared to psoriasis, while female patients also carried an increased risk of ischemic strokes and cardiac arrest.

Many of the previously published studies on this topic are restricted by their population cohorts (e.g., population size and/or monoethnicity), derivation from a single institutional center, or unclear exclusion criteria [20,21,24,29]. Our use of TriNetX facilitates the collection of robust population data, drawing from over 96 million patients across 64 health care organizations in the United States collaborative network. This allows for real-world comparisons among larger patient cohorts with existing comorbidities, thereby increasing generalizability. Additionally, our study required patients to have two independent HS diagnosis codes, as well as a long follow-up time, in order to minimize misdiagnosis or miscoding. Conversely, most previous studies only require one HS diagnosis coding and could lead to the inclusion of misdiagnoses [20,22,23,24,29]. Furthermore, very few studies have utilized another well-known inflammatory disease, such as psoriasis, as a control cohort [20]. To our knowledge, only one study has used a large, multi-center database such as TriNetX to investigate the association between HS and risk of CV disease. However, this study was limited to 5-year outcomes, only used healthy controls for comparison, does not include systemic immunological agents in the matching criteria, and only required one ICD-10 coding for HS, which may have included patients falsely diagnosed with HS at an initial evaluation [23].

It is important to note that although the risk of CV mortality rates in the United States (US) declined from 2010 to 2019, this trend reversed in 2020 [30,31,32,33]. This reversal signifies nearly a decade of lost progress in reducing CV mortality rates in the US, with age-adjusted mortality rates in 2022 reaching a level not previously observed between 2010 and 2021. Furthermore, it is predicted that the prevalence of CV disease will continue to increase over the next 30 years in the US [34]. Additionally, the US has been shown to have a greater prevalence of CV diseases, such as heart disease, stroke, and hypertension, than European countries [35]. This suggests patients with HS in the US are at even higher risk of CV disease than previously described, warranting this large-scale analysis of the US population. These data also explain the need to periodically update the risk faced by HS patients, both to identify potential therapeutic areas to improve upon and to describe at-risk populations.

The limitations of this study include the use of ICD-10, CPT, and ATC codes to identify patient diagnoses, which can be affected by misdiagnosis or miscoding. Furthermore, we did not match for systemic cardioprotective (e.g., beta blockers, aspirin) medications between cohorts, which may be a potential confounder. The mean age of our study population was also low, which may explain the relatively low number of adverse outcomes observed. Due to the observational study design, we cannot establish causality. TriNetX does not allow stratification based on cause of death; therefore, we are not able to assess cardiovascular disease-specific mortality. Finally, due to the nature of the dataset, we are unable to stratify by HS severity; we attempted to mitigate this limitation by matching cohorts for the use of immunological agents.

Given the availability of retrospective data available on this topic, future works could focus on prospective cohort studies aimed at mitigating the risk of cardiovascular disease observed herein. Additionally, future projects could analyze the impact of HS severity in risk elevation, as more severe disease, and therefore inflammation, may greatly increase risk. Relatedly, another area of investigation could evaluate the potential impact of different treatment modalities on the risk of cardiovascular disease and other highly morbid outcomes.

Our analysis demonstrates an elevated risk of a myriad of adverse cardiovascular outcomes and all-cause mortality in patients with HS. These results were independent of other factors associated with cardiovascular disease risk, and most remained elevated when comparing HS patients to those with psoriasis. Our findings highlight the potential benefit of screening for and managing modifiable cardiovascular risk mediators in HS patients. Further additional studies could investigate the potential underlying molecular mechanisms in HS patients or could assess the clinical consequences of this association.

## Figures and Tables

**Table 1:** Baseline characteristics of study population after matching for cardiovascular disease risk factors.

Characteristic	Cases (n = 61,200)	Controls (n = 61,200)	*p*-Value
**Demographics, n (%)**			
Current Age, mean (SD)	42 (13.4)	42 (13.4)	0.97
Female	46,289 (75.6)	46,292 (75.6)	0.98
Male	14,533 (21.5)	14,908 (24.4)	0.97
White	26,459 (43.2)	26,467 (43.2)	0.96
Black	20,722 (33.9)	20,743 (33.9)	0.90
Unknown race	9543 (15.6)	9529 (15.6)	0.91
BMI ≥ 30	20,979 (34.3)	20,981 (34.3)	0.99
**Comorbidities, n (%)**			
Liver disease	1824 (3.0)	1629 (2.7)	0.13
Chronic lower respiratory disease	8984 (14.7)	8782 (14.4)	0.10
Hyperlipidemia	7118 (11.6)	6936 (11.3)	0.25
Nicotine dependence	8243 (13.5)	8260 (13.5)	0.89
Alcohol-related disorders	1169 (1.9)	1104 (1.8)	0.17
Diabetes mellitus	5578 (9.1)	5406 (8.8)	0.09
Immunological agents	15,912 (26.0)	15,763 (25.7)	0.33

Abbreviations: SD, standard deviation; n, number; BMI, body mass index.

**Table 2:** Absolute and relative risks in HS patients compared to healthy controls ^a^.

Outcome	No. of Events, HS Group (%) ^b^	No. of Events, HC Group (%) ^b^	RR (95% CI)	*p*-Value
MI	850 (1.39)	412 (0.67)	2.06 (1.83–2.32)	<0.0001
Ischemic CVA	717 (1.17)	443 (0.72)	1.62 (1.44–1.82)	<0.0001
Hemorrhagic CVA	178 (0.29)	106 (0.17)	1.68 (1.32–2.13)	<0.0001
HF	1850 (3.02)	837 (1.37)	2.21 (2.04–2.40)	<0.0001
Cardiac Arrest	217 (0.36)	116 (0.19)	1.87 (1.49–2.34)	<0.0001
MACEs	2970 (4.86)	1523 (2.49)	1.95 (1.84–2.07)	<0.0001
VTEs	1708 (2.79)	904 (1.48)	1.89 (1.74–2.04)	<0.0001
CAD	2136 (3.49)	1237 (2.02)	1.73 (1.60–1.84)	<0.0001
All-cause Mortality	1473 (2.40)	573 (0.94)	2.57 (2.34–2.83)	<0.0001

^a^ Risks were analyzed after matching for common and significant cardiovascular disease risk factors such as hypertension, dyslipidemia, and nicotine use. ^b^ Based on a population of 61,200 individuals after matching. Abbreviations: HS, hidradenitis suppurativa; HC, healthy controls; No., number; RR, relative risk; CI, confidence interval; MI, myocardial infarction; CVA, cerebrovascular accident; HF, heart failure; MACEs, major adverse cardiac events; VTEs, venous thromboembolisms; CAD, coronary artery disease.

**Table 3:** Risk of cardiovascular disease in HS patients, stratified by sex.

	Male HS vs. Male Healthy Controls (n = 14,535)				Female HS vs. Female Healthy Controls (n = 50,850)			
Outcome	Absolute Risk, HS, n (%)	Absolute Risk, HC, n (%)	Relative Risk (95% CI)	*p*-Value	Absolute Risk, HS, n (%)	Absolute Risk, HC, n (%)	Relative Risk (95% CI)	*p*-Value
MI	232 (1.6)	138 (1.0)	1.67 (1.36–2.06)	<0.0001	437 (0.9)	236 (0.5)	1.85 (1.58–2.17)	<0.0001
Ischemic CVA	141 (1.0)	114 (0.8)	1.23 (0.96–1.57)	0.096	421 (0.8)	260 (0.5)	1.62 (1.38–1.89)	<0.0001
Hemorrhagic CVA	49 (0.3)	28 (0.2)	1.75 (1.10–2.78)	0.017	96 (0.2)	70 (0.1)	1.37 (1.01–1.86)	0.0437
HF	483 (3.3)	242 (1.7)	1.99 (1.71–2.32)	<0.0001	963 (1.9)	486 (1.0)	1.98 (1.77–2.20)	<0.0001
Cardiac Arrest	74 (0.5)	57 (0.4)	1.30 (0.92–1.83)	0.1375	113 (0.2)	76 (0.1)	1.49 (1.11–1.99)	0.0071
MACEs	736 (5.1)	434 (3.0)	1.68 (1.49–1.89)	<0.0001	1517 (3.0)	836 (1.7)	1.80 (1.66–1.96)	<0.0001
VTEs	327 (2.3)	175 (1.2)	1.86 (1.55–2.23)	<0.0001	1000 (2.0)	516 (1.0)	1.93 (1.74–2.15)	<0.0001
CAD	459 (3.2)	291 (2.0)	1.57 (1.35–1.82)	<0.0001	712 (1.4)	397 (0.8)	1.80 (1.59–2.04)	<0.0001
All-cause Mortality	608 (4.2)	280 (1.9)	2.17 (1.89–2.49)	<0.0001	916 (1.8)	548 (1.1)	1.67 (1.50–1.86)	<0.0001

Abbreviations: HS, hidradenitis suppurativa; HC, healthy controls; RR, relative risk; CI, confidence interval; CVA, cerebrovascular accident; HF, heart failure; MACEs, major adverse cardiac events; VTEs, venous thromboembolisms; CAD, coronary artery disease.

**Table 4:** Baseline characteristics of study and psoriasis populations after matching for cardiovascular disease risk factors.

Characteristic	Cases (n = 48,163)	Psoriasis Controls (n = 48,163)	*p*-Value
**Demographics, n (%)**			
Current Age, mean (SD)	44.3 (13.8)	44.6 (14.1)	0.0013 ^a^
Female	34,254 (71.1)	34,296 (71.2)	0.76
Male	12,304 (25.5)	12,346 (25.6)	0.76
White	27,085 (56.2)	27,091 (56.2)	0.97
Black	8480 (17.6)	8672 (18.0)	0.11
Unknown race	8482 (17.6)	8454 (17.5)	0.81
BMI ≥ 30	12,898 (26.8)	13,088 (27.2)	0.17
**Comorbidities, n (%)**			
Liver disease	1493 (3.1)	1378 (2.9)	0.02 ^a^
Chronic lower respiratory disease	5989 (12.4)	6058 (12.6)	0.50
Hyperlipidemia	5796 (12.0)	5845 (12.1)	0.63
Nicotine dependence	5513 (11.5)	5768 (12.0)	0.01 ^a^
Alcohol-related disorders	918 (1.9)	904 (1.9)	0.74
Diabetes mellitus	3972 (8.2)	4097 (8.5)	0.15
Immunological agents	11,092 (23.0)	11,019 (22.9)	0.58

^a^ This value is still considered appropriately matched due to having a standardized mean difference of <0.1, which is also statistically demonstrative of a small difference.

**Table 5:** Absolute and relative risk in HS patients compared to psoriasis patients ^a^.

Outcome	No. of Events, HS Group (%) ^b^	No. of Events, Psoriasis Group (%) ^b^	RR (95% CI)	*p*-Value
MI	742 (1.54)	566 (1.17)	1.31 (1.17–1.47)	<0.0001
Ischemic CVA	616 (1.28)	614 (1.27)	1.04 (0.93–1.16)	0.9557
Hemorrhagic CVA	156 (0.32)	148 (0.31)	1.05 (0.84–1.31)	0.6847
HF	1564 (3.25)	1253 (2.60)	1.24 (1.15–1.34)	<0.0001
Cardiac Arrest	187 (0.39)	163 (0.34)	1.04 (0.84–1.28)	0.7441
MACEs	2542 (5.28)	2193 (4.56)	1.16 (1.09–1.22)	<0.0001
VTEs	1367 (2.84)	1208 (2.51)	1.17 (1.08–1.27)	<0.0001
CAD	1924 (4.00)	1744 (3.62)	1.10 (1.03–1.18)	0.0025
All-cause Mortality	1303 (2.71)	997 (2.07)	1.38 (1.27–1.50)	<0.0001

^a^ Risks were analyzed after matching for common and significant cardiovascular disease risk factors such as hypertension, dyslipidemia, and nicotine use. ^b^ Based on a post-match population of 48,163 patients in each cohort. Abbreviations: HS, hidradenitis suppurativa; RR, relative risk; CI, confidence interval; MI, myocardial infarction; CVA, cerebrovascular accident; HF, heart failure; MACEs, major adverse cardiac events; VTEs, venous thromboembolisms; CAD, coronary artery disease.

## Data Availability

Data are available through the online dataset: https://live.trinetx.com/ (accessed 10 December 2024).
